# The rising SARS‐CoV‐2 JN.1 variant: evolution, infectivity, immune escape, and response strategies

**DOI:** 10.1002/mco2.675

**Published:** 2024-07-29

**Authors:** Yishan Lu, Danyi Ao, Xuemei He, Xiawei Wei

**Affiliations:** ^1^ State Key Laboratory of Biotherapy West China Hospital Sichuan University Sichuan People's Republic of China

**Keywords:** immune evasion, JN.1 variant, response strategies, vaccine

## Abstract

The JN.1 variant of COVID‐19 has emerged as the dominant strain worldwide since the end of 2023. As a subclade of the BA.2.86 variant, JN.1 harbors a unique combination of mutations inherited from the BA.2.86 lineage, notably featuring the novel L455S mutation within its receptor‐binding motif. This mutation has been linked to increased transmissibility and enhanced immune evasion capabilities. During the rise of JN.1, evidence of resistance to various monoclonal antibodies and reduced cross‐neutralization effects of the XBB.1.5 vaccine have been observed. Although the public health threat posed by the JN.1 variant appears relatively low, concerns persist regarding its evolutionary trajectory under immune pressure. This review provides a comprehensive overview of the evolving JN.1 variant, highlighting the need for continuous monitoring and investigation of new variants that could lead to widespread infection. It assesses the efficacy of current vaccines and therapeutics against emerging variants, particularly focusing on immunocompromised populations. Additionally, this review summarizes potential vaccine advancements and clinical treatments for COVID‐19, offering insights to optimize prevention and treatment strategies. This review thoroughly evaluates the JN.1 variant's impact on public health and its implications for future vaccine and therapeutic development, contributing to ongoing efforts to mitigate the risk of virus transmission and disease severity.

## 1 INTRODUCTION

The COVID‐19 pandemic, caused by severe acute respiratory syndrome coronavirus 2 (SARS‐CoV‐2), has represented a paramount global health crisis over the past 5 years.^1^ Since its outbreak in December 2019, more than 775 million confirmed cases and 7.04 million deaths have been reported worldwide.^2^, ^3^ Attributed to the development of relevant drugs and vaccines, coupled with proactive management and control measures implemented by the World Health Organization (WHO) and various countries, the COVID‐19 pandemic has transitioned from a public health emergency of international concern to a stage of sustained management.^3^ Nevertheless, due to the extensive spread and evolution of the virus and alterations in population immunity, the genome of SARS‐CoV‐2 has undergone mutations, leading to the emergence of new variants.^4^, ^5^ These mutations influence the biological characteristics of new viruses, thereby affecting their infectivity, transmissibility, and the efficacy of vaccines and therapeutic drugs.^6^ Therefore, to expedite research and facilitate public health risk assessment of variants, the WHO designates different strains as variants of concern (VOCs), variants of interest (VOIs), and variants under monitoring (VUMs). As of April 2024, there are five VOCs named Alpha (B.1.1.7), Beta (B.1.351), Delta (B.1.617.2), Gamma (P.1), and Omicron (B.1.1.529). The current prevalence VOIs include XBB.1.5, XBB.1.16, EG.5, BA.2.86, and JN.1, while the VUMs contain JN.1.7, JN.1.18, KP.2, and KP.3.^7^ Notably, JN.1, the latest VOI, has widely spread across multiple regions, supplanting other variants and instigating new waves of infections.^8^ As of February 5, 2024, 79,107 JN.1 sequences from 94 countries have been submitted to GISAID, accounting for 89.0% of the globally available sequences from January 22 to 28, 2024.^7^ In May 2024, JN.1 has been reported by 130 countries, which is still the most reported variant of interest, accounting for 54.3% of sequences.^9^

Descending from the Omicron variant BA.2.86 lineage, JN.1 harbors only one additional mutation site compared to BA.2.86 within the receptor binding domain (RBD) region of the spike protein. This mutation significantly alters the binding affinity of JN.1 to the angiotensin‐converting enzyme 2 (ACE2) receptor and considerably enhances its immune evasion ability.^10^, ^11^, ^12^, ^13^ Several studies have proven that JN.1 had heightened transmissibility relative to BA.2.86, showing stronger resistance to the newest monovalent XBB.1.5 vaccine. Hence, monitoring new emerging variants of SARS‐CoV‐2 is crucial to address the public health challenge posed by COVID‐19. In this article, we provide an overview of the epidemiology, clinical characteristics, and immune evasion ability of JN.1, drawing insights from current reports. Additionally, we assess the efficacy of existing vaccines against JN.1 variant infections and propose strategies for vaccine development in the post‐epidemic period.

## 2 VIROLOGICAL CHARACTERISTICS OF JN.1

### 2.1 Identification and timeline of JN.1 transmission

The emergence of Omicron variants, characterized by numerous mutations within RBD, has sparked an unparalleled surge in infections globally. Several VOIs, including XBB.1.5 and BA.2.86, have arisen from the BA.2 lineage of the Omicron variant. BA.2.86 was first identified in August 2023 and exhibited notable differences from BA.2 and XBB.1.5 lineages, with 31 and 27 mutations in the spike protein, respectively.^14^ Studies have highlighted a substantially increased binding affinity of BA.2.86 to the ACE2 receptor and multiple unusual mutations in the N‐terminal domain, which may change the antigenicity of BA.2.86.^15^, ^16^ Although BA.2.86 did not achieve the widespread dissemination observed with other variants, studies have emphasized the considerable transmission potential of it. As the virus undergoes further mutations, the new variant JN.1, a descendant of BA.2.86, has been noted for its exceptional transmissibility.

**FIGURE 1:**
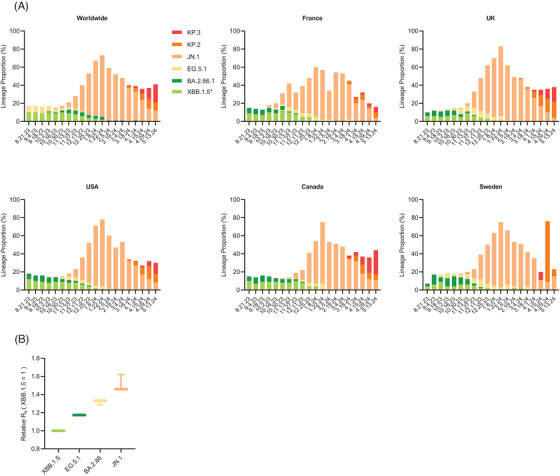
The lineage proportion of Omicron subvariants. (A) Proportion of XBB.1.5, EG.5.1, BA.2.86, JN.1, KP.2, and KP.3 that moved from August 21, 2023 to May 13, 2024 globally and in other countries (France, UK, USA, Canada, and Sweden). Data were acquired from GISAID. (B) Relative *R*_e_ of XBB.1.5, EG.5.1, BA.2.86, and JN.1.

The first confirmed case of JN.1 (BA.2.86.1.1) was discovered in France in August 2023.^17^ Subsequently, JN.1 rapidly spread across various countries and regions, such as the USA, the UK, France, Canada, and Sweden, eventually supplanting XBB.1.5 and other strains to become the predominant variant globally.^18^, ^19^ According to the U.S. Centers for Disease Control and Prevention (CDC), the initial prevalence of JN.1 in the USA was less than 0.1% at the end of October.^20^ However, by late December, the proportion of JN.1 quickly climbed to 44% (Figure 1A), marking it the fastest‐growing variant in the USA. At the end of January 2024, the JN.1 proportion in the UK, France, and Sweden had reached 77%, 55%, and 72%, respectively (Figure 1A). Meanwhile, the global prevalence of JN.1 exceeded 60% (Figure 1A), reaching its peak in the transmission stage. At present, with the evolution of JN.1, its descendants KP.2 and KP.3 have been identified in multiple regions and shown increasing transmissibility. As of May 13, 2024, the global proportions of KP.2 and KP.3 have reached 9% and 20%, respectively (Figure 1A).

The reproductive number (*R*_e_) is designated to measure the average number of infected cases in populations during the epidemic circulation. After conducting a transmissibility analysis on JN.1 and several previously popular variants, it was determined that when the relative *R*_e_ value of XBB.1.5 was set at 1, the relative *R*_e_ of EG.5.1, BA.2.86, and JN.1 were 1.175, 1.319, and 1.512, respectively (Figure 1B). Notably, the relative *R*_e_ of JN.1 was 1.1 times higher than that of BA.2.86 and 1.2 times that of EG.5.1, indicating a markedly more robust transmission capability of JN.1. This finding is consistent with the recent pandemic surge attributed to JN.1.

### 2.2 Clinical symptomatology and susceptible population

Currently, limited reports indicate that BA.2.86 or JN.1 will lead to different clinical manifestations from other Omicron subvariants. Symptoms induced by these variants are similar and may include sore throat, fever, cough, shortness of breath, nasal congestion, fatigue, headache, muscle aches, altered sense of smell, and other discomforts.^21^ The severity and manifestation of these symptoms partly depend on the immunity to vaccination status and previous infections of individuals.

This disease appears to affect men more frequently than women, with a notable inclination toward the elderly, especially those aged over 60 years.^22^ Studies on the elderly population have shown that immunosenescence and comorbidities may impair the effectiveness of vaccines, rendering older adults with multiple comorbidities more susceptible to the virus.^23^ According to the CDC, hospitalization and severe disease rates among infants, young children, and the elderly have increased during the JN.1 infection crisis. Older adults are prone to experiencing complications after being infected with COVID‐19, leading to a higher incidence of severe illness. Due to the prevalence of other seasonal respiratory diseases such as mycoplasma pneumonia, influenza, and respiratory syncytial virus (RSV), JN.1 exacerbates the hospitalization in infants and young children with low immunity, resulting in a surge in the rate of severe disease.^24^ Notably, no deaths have been reported as a direct result of this new Omicron variant.^25^ Although the WHO opines that the spread of the JN.1 variant is improbable to increase the burden on the public health system, it is still imperative to remain vigilant regarding infections in special populations.^26^

### 2.3 Serotype classification and antigenic relationships of JN.1

The diminished cross‐neutralization observed among different variants has resulted in a decline in the efficacy of current therapeutic antibodies and vaccines.^27^ This reduction in cross‐neutralization is also a defining characteristic of serotype divergence.^28^ The serotype classification of SARS‐CoV‐2, which is closely related to medicine development, is helpful in informing prevention and control measures.^29^ Until now, five serotypes have been delineated based on the antigenicity of RBD and cross‐neutralization.^30^ Research investigating the neutralizing capacity of BA.2.86 antisera against various variants, such as XBB, indicated a reduction in neutralization titers by 1−2 orders of magnitude. Analysis of homologous and heterologous titer suggested a substantial diminishment in the cross‐reactivity of BA.2.86 with other serotypes, classifying it as a distinct serotype VI.^31^, ^32^ Serotype studies on JN.1 are limited, as JN.1 inherits characteristics from BA.2.86, so the difference in bidirectional cross‐reactivity between JN.1 and XBB or other lineages may be more distinct.

Remarkably, serotype classification correlates with phylogenetic analysis of viral variants.^33^ Although JN.1, BA.2.86, XBB.1.5, and other variants belong to the ancestral BA.2, their positions on the phylogenetic tree indicate that the evolutionary branches of BA.2.86 and JN.1 are entirely distinct from the XBB lineage. According to the antigen map, JN.1 aligns with BA.2.86, which is located at a different distinction, clustering further away from other variants, including wild types, XBB, BA.2, and BA.5 lineages, thereby demonstrating conspicuous antigenic drift.^14^, ^34^ Based on multiple studies, it is speculated that JN.1 may share a similar serotype with BA.2.86, exhibiting significant antigenic differences from other prevalent lineages.

### 2.4 Mutation characteristics of JN.1 spike

Although the evolutionary processes of variants are varied, mutations in RBD of XBB, BQ.1.1, and other variants have displayed a trend of convergent evolution.^16^ However, BA.2.86 and its descendant JN.1, with a series of novel and distinctive alterations in the spike, are contrary to the substitution and stable accumulation observed in the XBB lineage.^13^

**FIGURE 2:**
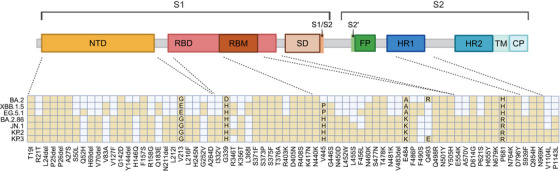
Spike protein mutations in prevailing variants. Mutations of BA.2, XBB.1.5, EG.5.1, BA.2.86, JN.1, KP.2, and KP.3 on the spike glycoprotein. The yellow color denotes the existing mutations, while the gray color represents absent mutations. CP, cytoplasm domain; FP, fusion peptide; HR1, heptad‐repeat sequences 1; HR2, heptad‐repeat sequences 2; NTD, N‐terminal domain; RBD, receptor‐binding domain; RBM, receptor‐binding motif; S2ʹ, S2ʹ protease cleavage site; TM, transmembrane domain.

Compared to the XBB.1.5 lineage, the latest vaccine strains with mutations in JN.1 are located in the spike protein and exhibit unique selective changes. These mutations include 27 amino acid alterations inherited from BA.2.86 and an additional L455S mutation in the receptor‐binding motif (RBM) of the RBD.^35^ Studies have demonstrated that the immune evasion capability of JN.1 is closely linked to these new mutations, while ACE2 binding affinity is more associated with the virus's transmission adaptability within the population.^13^, ^15^ Since L455 is crucial for binding to human ACE2, it is hypothesized that the L455S mutation in JN.1 enhances its immune evasion capability at the expense of reduced binding affinity to ACE2.^36^ Other mutations in the RBD, such as E554K, N450D, K356T, L452W, A484K, V483del, and V445H, also contribute to evasion from multiple monoclonal antibodies and various vaccines, thereby enhancing the immune evasion potential of JN.1.^11^, ^37^ These findings suggest a potential reduction in the effectiveness of the XBB.1.5 vaccine. Moreover, the KP.3 variant has acquired a new mutation, Q493E, in the RBD region, which may facilitate the transmission of KP.3^38^, ^39^ (Figure 2).

## 3 THE IMMUNE RESISTANCE OF THE JN.1VARIANT

Although the emergency phase of the COVID‐19 pandemic has been declared over, the spread and infection of the SARS‐CoV‐2 virus persist, resulting in the mutations of novel variants. These mutations help the virus to escape from the protection acquired through natural infection, vaccines, and drugs, thus continuing to threaten global public health security.^40^, ^41^ Consequently, it is imperative to comprehensively evaluate the therapeutic efficacy of available drugs and vaccines against variants such as BA.2.86 and JN.1, especially the currently updated XBB series of vaccines.

### 3.1 Sensitivity of the JN.1 variant to prevailing antiviral drugs and monoclonal antibodies

According to related studies, the approved oral antiviral drugs for SARS‐CoV‐2, including Paxlovid, Molnupiravir, and Remdesivir, remained highly effective to BA.2.86, JN.1 and variants derived from XBB.^13^ Moreover, no evidence suggested that BA.2.86 and JN.1 were resistant to available antiviral drugs.

In addition to antiviral drugs, Omicron variants escaped the neutralizing effect of most monoclonal antibodies.^42^ Monoclonal antibodies targeting the spike protein are classified into four types (I, II, III, and IV) based on the different conformations of the RBD.^43^, ^44^ Compared with BA.2.86, the JN.1 variant only adds a single L455S mutation, predominantly situated within the epitope of class I antibodies.^16^, ^45^ Virus neutralization data indicated that the addition of the L455S improved the ability of JN.1 to evade class I antibodies and enhanced the resistance of humoral immunity, compensating for the vulnerability of BA.2.86 against class I antibodies.^11^ Furthermore, mutations such as N450D, K356T, L452W, A484K, V483del, and V445H on RBD contribute to the enhanced immune evasion of BA.2.86, among which K356T, L452W, and P445H escape most antibodies in class III, while A484K and V483del evade most class II monoclonal antibodies.^46^ JN.1, the derivative of BA.2.86, exhibits similar characteristics. Additionally, studies have illustrated that the E554K mutation carried by both BA.2.86 and JN.1, situated on the binding interface of SD1‐targeting antibodies, enables JN.1 to evade SD1‐targeting neutralizing antibodies (Nabs), such as S3H3.^47^ Studies evaluating the sensitivity of D614G, XBB, EG.5.1, BA.2.86.1, and JN.1 to the class III monoclonal antibody Sotrovimab (S309) found that, aside from the ancestral D614G, Sotrovimab effectively neutralizes XBB and EG.5.1 but exhibits minimal neutralizing activity against BA.2.86.1 and even loses antiviral efficacy against JN.1.^11^, ^48^ As for the descendants of JN.1, KP.2, and KP.3 can also abolish S309 binding because of the possessed D339H mutation that creates a steric hindrance at the center of the class III antibody epitope.^49^ Comparative analysis of the neutralizing ability data of various monoclonal antibodies against XBB.1.5, EG.5, BA.2.86, and JN.1 revealed that BA.2.86 and JN.1 indeed exhibited widespread resistance to many types of monoclonal antibodies (Table 1).^11^, ^12^ Moreover, BA.2.86.1 and JN.1 were entirely resistant to many previously approved cocktail therapies, such as Ronapreve (Imdevimab + Casirivimab) and Evusheld (Cilgavimab + Tixagevimab).^50^ Interestingly, the SA55 monoclonal antibody retained its neutralizing efficacy against XBB.1.5, EG.5, BA.2.86, and JN.1.^51^ Since SA55 belongs to the RBD class IV/I monoclonal antibodies, further investigation is needed.

**TABLE 1:** Nabs resistance against circulating variants.

Pseudovirus IC50 (μg/mL)	S3H3 (SD1)	Omi‐42 (RBD class I)	S309 (RBD class III)	LY‐CoV1404 (RBD class III)	COV2‐2196 + COV2‐2130 (RBD class II + RBD class III)	REGN10933 + REGN10987 (RBD class I + RBD class III)	SA55 (RBD class IV/I)	Reference
XBB.1.5	0.036	0.004	0.97	>10	>10	>10	0.008	^11^, ^12^
EG.5	0.067	0.038	0.88	NA	NA	NA	0.008	^11^, ^12^
BA.2.86	>10	0.004	1.89	>10	>10	>10	0.006	^11^, ^12^
JN.1	>10	0.029	2.3	NA	NA	NA	0.009	^11^, ^12^

*Note*: IC50 (μg/mL) of approved or candidate neutralizing monoclonal antibody drugs targeting RBD or SD1 on spike against XBB.1.5, EG.5, BA.2.86, and JN.1 pseudoviruses.

Abbreviation: IC50, half‐maximal inhibitory concentration.

### 3.2 Current COVID‐19 vaccine effectiveness against the JN.1 variant

With the genetic changes and volatile evasion capabilities of SARS‐CoV‐2, it is crucial to evaluate the immunity induced by the current vaccines.^52^, ^53^ Wang and coworkers^27^ and Link‐Gelles et al.^54^ have shown limited efficacy of SARS‐CoV‐2 wild‐type monovalent and bivalent (wild type + Omicron BA.5) mRNA vaccines against emerging Omicron subvariants. Therefore, more attention should be directed toward assessing the efficacy of the latest XBB.1.5 vaccines.

In a recent study, neutralizing antibody levels against different variants were evaluated following the booster of the XBB.1.5 monovalent mRNA vaccine. The study involved individuals who initially received three doses of wild‐type monovalent, followed by a fourth dose of BA.5 bivalent vaccine, and then a fifth dose of the XBB.1.5 monovalent vaccine or experienced XBB breakthrough infection after the fourth vaccination. It is noticed that both the XBB.1.5 monovalent mRNA vaccine and the XBB.1.5 breakthrough infection led to elevated neutralizing antibodies against circulating virus variants, such as XBB.1.5, EG.5.1, and JN.1.^34^ Interestingly, the neutralizing antibody efficacy induced by the XBB.1.5 monovalent vaccine was comparable to that of the XBB.1.5 breakthrough infection. In essence, XBB breakthrough infection triggers a cross‐protective response, resulting in moderate neutralization of JN.1.^13^

Additionally, in another study, after the fifth dose of XBB.1.5 monovalent mRNA vaccine (mRNA‐1273.815), antibody titers against XBB.1.5, EG.5.1, BA.2.86, and JN.1 increased by 10−17 times. However, compared to the neutralizing ability against the wild type, neutralizing antibody titers were reduced by 4.4, 11.7, 11.3, and 26.1 times, respectively (Table 2).^34^, ^55^, ^56^ Notably, the JN.1 strain showed the most significant decline, indicating the evasion properties of JN.1.^55^ Moreover, concerning the antigen map, vaccination with the XBB.1.5 monovalent vaccine significantly reduced the antigenic distance between D614G and other SARS‐CoV‐2 variants, demonstrating a sufficient potency.^31^ But JN.1 remains distant from XBB.1.5 on the map, highlighting its antigenically distinct and potential evasion from the XBB.1.5 monovalent vaccine.^31^

**TABLE 2:** Neutralization of SARS‐CoV‐2 sub‐lineages by vaccination sera.

Geometric mean ID50 titers (GMT)	D614G	BA.5	XBB.1.5	EG.5.1	JN.1	BA.2.86	Total cases	Male (numbers)	Female (numbers)	Age (years), median (range)	Number of vaccine doses	COVID‐19 infection times and type	Reference
Three doses of WT monovalent vaccine + BA.5 bivalent vaccine + XBB.1.5 monovalent vaccine	6088	3121	561	588	196	NA	16	5	11	51.8 (36‒65)	5	NA	34
Three doses of WT monovalent vaccine + BA.5 bivalent vaccine + XBB infection	10,767	7519	1693	1575	379	NA	19	3	16	48.6 (33‒77)	4	NA	34
Two doses of primary series + 3rd original COVID‐19 vaccine + 4th dose of bivalent (Omicron BA.4/BA.5 plus original strain) vaccine + 5th dose of monovalent XBB.1.5 spike mRNA vaccine (mRNA‐1273.815)	12,191	11,231	2711	1042	466	1077	50	20	30	54.5 (21‒84)	5	NA	55
Two doses of primary series + 3rd original COVID‐19 vaccine + 4th dose of bivalent (Omicron BA.4/BA.5 plus original strain) vaccine + 5th dose of bivalent mRNA vaccine‐XBB.1.5 and BA.4/5 spike mRNAs (mRNA‐1273.231)	9067	11,037	2060	NA	NA	NA	51	20	31	48 (24‒82)	5	NA	55
Three doses of inactivated vaccines + BA.5/BF.7 breakthrough infection + reinfection with XBB virus	1072	3343	681	857	725	928	20	7	13	32 (18‒69)	3	2, BA.5/BF.7 or XBB	56
Three doses of inactivated vaccines + BA.5/BF.7 breakthrough infection + trivalent XBB vaccine (WSK‐V102C)	2833	9151	2576	1335	2567	2536	11	5	6	50.45 (25‒73)	4	1, BA.5/BF.7 or XBB	56

mean ID50 titers (GMT) of different vaccines against D614G and Omicron subvariants, the relative information of subjects involved in experiments.

Another XBB.1.5 trivalent mRNA (WSK‐V102C) vaccine showed adequate efficacy against a range of variants, including JN.1. Research data revealed that after administering the fifth dose of XBB.1.5 trivalent mRNA vaccine, the geometric mean titers (GMTs) for BA.2.86 and JN.1 were 2536 and 2567, which were compared to the GMT of 2833 against the D614G. Moreover, the trivalent vaccine elicited higher neutralizing titers than XBB breakthrough infection.^56^ Based on the above studies, it is evident that facing variants with robust evasion capabilities, such as JN.1, the effectiveness of various XBB.1.5 vaccinations remains to be further assessed.

## 4 STRATEGIES FOR OVERCOMING THE EVASION OF JN.1

### 4.1 Current recommendations for managing JN.1

During the COVID‐19 pandemic, universally accepted individual measures have been established to mitigate COVID‐19 transmission. These measures include proper mask wearing, frequent handwashing, adequate ventilation, avoidance of crowded spaces, and adherence to good hygiene practices.^57^ Such measures are also effective in combating the JN.1 infection. Moreover, continued monitoring and management of variants such as JN.1 remain paramount, as they play a pivotal role in curbing the spread of new variants.^58^

In addition, prioritizing boosters containing the latest variant antigens is advisable to enhance public immunity against COVID‐19. Vaccination can help prevent reinfection and reduce the severity and duration of symptoms.^59^ The previous analysis illustrated that the XBB.1.5 vaccines could provide varying levels of protective efficacy against the main circulating prevalence variants, including BA.2.86, JN.1, and others.^32^, ^34^, ^55^, ^60^ Therefore, the widespread administration of XBB.1.5 boosters, particularly among immunocompromised populations such as the elderly, infants, and young children, is essential to mitigate variant infections and reduce hospitalization and mortality rates.

### 4.2 Future directions on the vaccines and therapies development

Due to the genetic mutations and selection pressure of SARS‐CoV‐2, variants such as JN.1 are evolving toward heightened immune evasion capabilities and increased transmissibility, thereby diminishing the efficacy of the XBB.1.5 monovalent vaccine. In the long‐term fight against SARS‐CoV‐2, vaccination is still a crucial tool for disease control and the protection of public safety.^59^

Although current vaccines remain safe and effective, the continued development of virus variants and their transmissibility pose challenges to vaccine development. At present, research efforts are focused on developing COVID‐19 vaccines using various methods. Among these, approaches utilizing mucosal immunity, such as intranasal or oral mucosa delivery, hold promise. However, practical strategies for inducing and enhancing mucosal immunity require further exploration.^61^ In terms of vaccine delivery systems, nanoparticle platforms, particularly ferritin nanocages, have garnered attention due to their remarkable biological stability, biocompatibility, and ability to integrate multiple antigens.^62^ As for vaccine antigens, vaccines targeting conserved regions, such as the nucleocapsid protein, offer potential advantages over those targeting the frequently mutated S protein, as they can elicit robust T‐cell immunity.^63^ Moreover, targeting the SARS‐CoV‐2 RNA‐dependent RNA polymerase for vaccine‐specific recognition is also ongoing.^64^ In addition to administrating the newest boosters, vaccination against influenza and RSV is also recommended to prevent “triple” infections, as observed in US hospitals during the winter of 2023/2023.^65^

Furthermore, in response to the COVID‐19 pandemic, it has been recognized that current global public health systems are inadequately equipped to prevent severe infectious diseases.^66^ Thus, allocating additional resources to research aimed at clinical treatments is vital, especially for individuals at high risk of severe illness or complications, or for whom vaccination alone may not suffice to safeguard their lives.^67^ Notably, passive immunotherapy strategies have emerged as crucial components of clinical therapies against COVID‐19, showing promising efficacy in virus clearance, syndrome attenuation, and tissue repair.^68^ These strategies encompass monoclonal antibody‐based treatment, ACE2 decoy receptor, T‐cell‐based therapies (SARS‐CoV‐2‐specific T‐cell therapy), extracellular vesicle‐based therapy, and NK‐cell‐based therapy.^63^ Moreover, mesenchymal/stromal cell‐based therapy and regulatory T‐cell‐based therapy represent immunomodulatory and regenerative approaches.^69^ It is foreseeable that continued research into these strategies will enhance global preparedness to confront future pandemic emergencies and public health challenges.

## 5 CONCLUSIONS

Until now, the emergence of new variants of COVID‐19 persists due to mutation and genetic recombination mechanisms. JN.1 was first detected in late 2023, showing divergence from its parent strain BA.2.86.^58^ With a unique mutation within the RBM, JN.1 rapidly sparked a global pandemic surge, demonstrating significant transmissibility and immune evasion capabilities.^36^, ^40^, ^41^ Symptoms associated with JN.1 infection share similarities with various variants of SARS‐CoV‐2.^21^ Although the emergence of JN.1 has not seriously burdened the global public health system, concerns persist due to the potential impact of emerging SARS‐CoV‐2 lineages on immunocompromised individuals and current therapies.^70^

According to the reports, approved antiviral drugs remain effective against BA.2.86 and JN.1.^13^ In the future, developing antiviral drugs are promising to control the SARS‐CoV‐2 variants infections since BA.2.86 and JN.1 display resistance to many monoclonal antibodies.^11^, ^12^, ^13^, ^42^, ^46^, ^48^ Even though the renewed monovalent XBB.1.5 vaccine still provides protection against JN.1, the induced neutralizing antibody titers show the most decline compared to the wild type.^32^, ^34^ Notably, the neutralizing titers induced by XBB.1.5 trivalent mRNA vaccine remain at a high level compared to the wild type, and higher neutralizing titers can be elicited compared to XBB breakthrough infection.^56^

In general, reducing virus transmission and mitigating the long‐term impact of viral infection are arduous challenges. Improved virus prevention and treatment strategies can broadly benefit individuals facing ongoing and emerging infectious diseases.^64^ Currently, one of the primary goals is to develop a vaccine that can provide broad and durable immunity against multiple COVID‐19 variants, promoting cross‐protection and ensuring high global accessibility. Effectively curbing the potential risks brought by the continuous evolution of SARS‐CoV‐2 variants, such as JN.1 and its derived strains, remains a crucial focus for researchers and governments worldwide.

## AUTHOR CONTRIBUTIONS

Xiawei Wei conceived the study and the manuscript. Yishan Lu and Danyi Ao wrote the paper. Yishan Lu and Xuemei He made Figure 1. Yishan Lu and Danyi Ao made Figure 2. All authors have read and approved the article.

## CONFLICT OF INTEREST STATEMENT

The authors declare they have no conflicts of interest.

## ETHICS STATEMENT

Not applicable.

## Data Availability

The data included in this study are available upon request from the corresponding author.
